# Clinical Outcomes Following Use of Tranexamic Acid in High Tibial Osteotomy: A Systematic Review

**DOI:** 10.7759/cureus.49556

**Published:** 2023-11-28

**Authors:** Ciara E O'Donnell, Hashim Dadah, Hamid Rahmatullah Bin Abd Razak, Adrian Wilson, Raghbir Khakha

**Affiliations:** 1 Medicine, King's College London, London, GBR; 2 General Surgery, Whipps Cross University Hospital, London, GBR; 3 General Surgery, King's College London, London, GBR; 4 Musculoskeletal Sciences, Duke-NUS Medical School, Singapore, SGP; 5 Orthopaedic Surgery, Sengkang General Hospital, Singapore, SGP; 6 Department of Sports and Exercise, University of Winchester, Winchester, GBR; 7 Orthopaedics, Guy's and St Thomas' NHS Foundation Trust, London, GBR

**Keywords:** meta-analysis, hto, haemoglobin, systematic review, surgery, orthopaedics, txa, tranexamic acid, tibia, high tibial osteotomy

## Abstract

This study aimed to evaluate the clinical outcomes following administration of tranexamic acid (TXA) in patients undergoing high tibial osteotomy (HTO) through a systematic review of current available evidence. A systematic database search of PubMed, Embase and Cumulative Index of Nursing and Allied Health Literature (CINAHL) was performed from inception up to December 2022, in accordance with the Preferred Reporting Items for Systematic Reviews and Meta-analyses (PRISMA). Inclusion criteria were (i) randomised control trials, cohort studies or case-control studies that had more than 10 patients; (ii) studies reporting outcomes after TXA administration, of any route, before or after HTO, compared to placebo, control and different doses or routes; and (iii) studies reporting blood loss, including haemoglobin (Hb) drop, estimated blood loss, transfusion requirement and complications. Case reports, reviews, abstracts, non-HTO studies, non-human studies and duplicates were excluded. A synthesized comparison of drain output, wound complications, transfusion requirement and pooled analyses of blood loss and Hb drop was performed. Eleven studies involving 974 patients were included. Nine studies had placebo comparison, and two used single-dose TXA versus multiple doses. All studies reported on postoperative hemoglobin and nine on blood loss. In the six TXA versus placebo studies reporting on total blood loss, the TXA group had a pooled, estimated standardised mean difference (SMD) in blood loss of -2.37 (95% confidence interval (CI) -3.67, -1.07; P = 0.0004). For the Hb drop, on postoperative days (PODs) one, two, and five, the SMDs were -0.97 (95% CI -1.19, -0.75; P < 0.00001) for POD1, -0.74 (95% CI -1.03, -0.46; P < 0.00001) for POD2 and -0.87 (95% CI -1.10, -0.64; P < 0.00001) for POD5. TXA administration in HTO significantly reduces perioperative blood loss. This can greatly improve recovery, reduce complications and shorten length of stay. This is especially pertinent given supply shortages of NHS blood resources.

## Introduction and background

The prevalence of knee osteoarthritis (OA) has doubled since the middle of the twentieth century, due to an increase in both life expectancy and body mass index (BMI) of the general population [1]. Medial opening wedge high tibial osteotomy (MOWHTO) has been gaining popularity as a therapy for OA, owing to the improved postoperative activity levels compared to unicompartmental or total knee arthroplasty surgery [2]. MOWHTO is primarily utilised as an early surgical intervention for younger patients with unicompartmental KOA [3], preventing progression of disease burden and reducing both the physical cost to patient and long-term financial costs to the NHS. As of 2018, OA and rheumatoid arthritis cost the NHS £10.2 billion per annum with an expectation to surpass £118.6 billion within the next 10 years [4]. Furthermore, such musculoskeletal conditions are the leading cause of disability, accounting for 30.5% of years lived with a disability [4].

While MOWHTO is a suitable procedure for patients with unicompartmental KOA, however, this procedure is not without risks. One of the most common risks include bleeding from the metaphyseal cancellous bone at the site of the osteotomy. This, combined with tissue manipulation, can potentially lead to significant blood loss, clinically demonstrated by a drop in the postoperative haemoglobin (Hb) [3]. This may cause an increase in both the length of stay (LoS) and the risk of blood transfusion postoperatively. To reduce risks of bleeding in MOWHTO surgery, several methods have been explored and researched. One of these is the use of tranexamic acid (TXA) intraoperatively through various routes of administration [5-15]. TXA is a fibrinolytic inhibitor, FDA-approved for menorrhagia and short-term use in haemophilia but with growing popularity and call for approval of intraoperative blood loss. While there are several studies that have been conducted to evaluate the safety and efficacy of TXA in preventing excessive blood loss following MOWHTO, there is no consensus with regard to its routine use.

Therefore, we aimed to systematically review the current literature with regard to the safety and efficacy of TXA in MOWHTO. Prior to the initiation of this study, we hypothesised that TXA would be a safe and efficacious intervention in MOWHTO.

This article was previously presented as a meeting abstract at ASIT 47th Annual Conference 2023, BOA Annual Conference 2023 and accepted for future presentation at BOTA Annual Conference 2023.

## Review

Information sources and study selection

**Figure 1:**
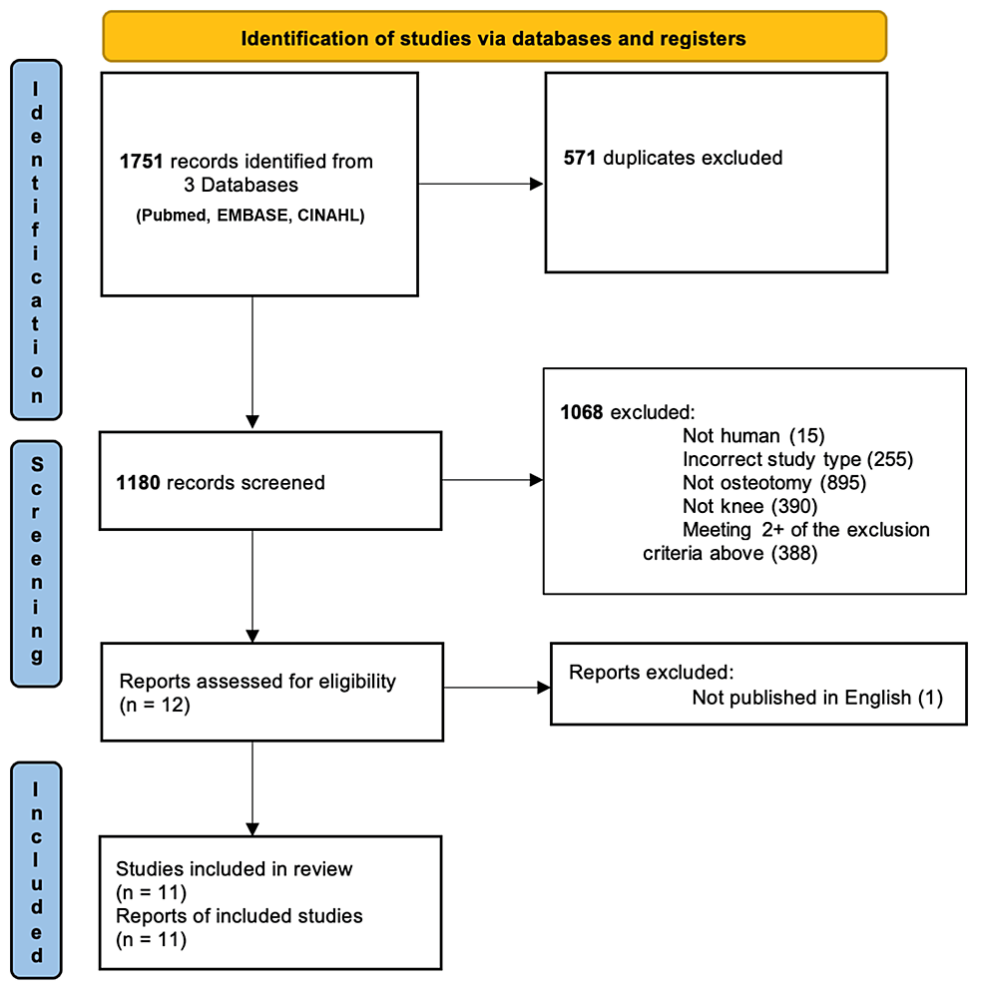
PRISMA flow diagram demonstrating study selection PRISMA: Preferred Reporting Items for Systematic Reviews and Meta-analyses

An electronic search was performed by two independent authors (C.O'D and H.D) in PubMed, Embase, and Cumulative Index of Nursing and Allied Health Literature (CINAHL) databases to identify all relevant studies, with the most recent search conducted in December 2022. The following search strategy was used to query citation titles and abstracts: ((osteotomy) OR (high tibial osteotomy) OR (hto) OR (knee) OR (proximal tibia)) AND ((tranexamic acid) OR (txa)). Our detailed search strategy for each of the databases is presented in Annex A. Reference lists of relevant systematic reviews were manually searched. This review was not registered on the International Prospective Register of Systematic Reviews (PROSPERO) database. The search workflow was in adherence to the Preferred Reporting Items for Systematic Reviews and Meta-Analyses (PRISMA) guidelines and is illustrated in Figure 1. To identify studies to be included in the final review, the articles were independently assessed by two authors (C.O'D and H.D), to determine eligibility for inclusion in the analysis. Any disagreements were resolved by consensus discussion among the authors. A total of 11 studies were included in the final review.

Eligibility criteria

The inclusion criteria adopted for the study selection were as follows: (i) randomised clinical trials (RCTs), cohort studies or case-control studies of at least 10 patients; (ii) studies reporting clinical outcomes following application of tranexamic acid via any route before or after MOWHTO, compared to placebo, control, different dosing or different routes of TXA administration; (iii) and studies reporting blood loss, measured by Hb drop, estimated blood loss, need for blood transfusion and complications. Case reports, review articles, published abstracts, non-MOWHTO operations, non-human studies and duplicate studies were excluded from this review. Articles in languages other than English were also excluded.

Quality assessment of the included studies

For RCTs, quality was assessed using the Cochrane Collaboration risk-of-bias version 2 (ROB 2) tool whose domains included (1) random sequence generation (selection bias), (2) allocation concealment (selection bias), (3) blinding of participants and personnel (performance bias), (4) blinding of outcome (detection bias), (5) incomplete outcome data (attrition bias), (6) selective reporting (reporting bias) and (7) other sources of bias. For non-RCTs, the Risk Of Bias In Non-Randomized Studies of Interventions (ROBINS-I) tool was used. The domains included (1) confounding bias, (2) bias in selection of participants, (3) bias in classification of interventions, (4) bias from deviation in intended interventions, (5) missing data bias, (6) outcome measurement bias and (7) bias in the selection of the reported result. For both ROBINS-I and ROB, the risk was deemed low, moderate or serious.

Data collection

All data from the texts, figures and tables of the included studies were extracted to Microsoft Excel spreadsheet software for analysis and review. The specific information extracted included the following: (i) study details, including study design and level of evidence; (ii) study population details, including number of patients, patient demographics and the size of the control group (if present); (iii) objective of study; (iv) TXA route of administration and dose given; (v) outcomes studied and results; and (vi) any reported complications. Outcomes evaluated in our systematic review were (i) blood loss (ml); (ii) transfusion requirements; (iii) Hb total decrease (g/dl); (iv) drain output; (v) hidden blood loss; and (vi) wound complications.

Statistical analysis

To evaluate the efficiency of TXA to reduce blood loss in HTO, either in comparison to placebo or as a multiple-dosing regimen, a fixed-effect model was used for the meta-analysis. The standardised mean difference (SMD) was used to compare the effects of the other variables (transfusion requirement, drain output, Hb drop and wound complications), with calculation of 95% confidence intervals (CIs) of the SMDs. An effect was considered statistically significant with a p-value less than 0.05. These other variables were chosen with the consideration of clinical importance and with reference to previous review articles.

Quality assessment of studies

The quality of the RCTs included in this study was assessed using the Cochrane Collaboration ROB-2 tool. The ROBINS-I tool was used in the evaluation of the quality of evidence for each outcome measure for the non-RCTs. The results of the quality assessment are illustrated in Table 1.

**Table 1:** Quality assessment of included studies References: [5-15] RCT: randomised controlled trial, ROB: risk of bias

RCTs	Random sequence generation	Allocation concealment	Blinding of participants and personnel	Blinding of outcome assessment	Incomplete outcome data	Selective reporting	Other bias	Overall ROB judgements
Kim et al., *Journal of Clinical Medicine* (2021) [8]	Low risk	Low risk	Low risk	Low risk	Low risk	Low risk	N/A	Low risk
Ni et al., *Archives of Orthopaedic and Trauma Surgery* (2021) [12]	Low risk	Unclear risk	Unclear risk	Low risk	Low risk	Low risk	Low risk	Low risk
Non- RCTs	Confounding	Selection of participants	Classification of interventions	Deviations from intended interventions	Missing data	Measurement of outcomes	Selection of reported results	Overall ROB judgements
Luo et al., *Acta Orthopaedica et Traumatologica Turcica* (2022) [10]	Moderate risk	Moderate risk	Low risk	Low risk	Low risk	Low risk	Low risk	Moderate risk
Petersen et al., *Archives of Orthopaedic and Trauma Surgery* (2022) [14]	Moderate risk	Moderate risk	Low risk	Low risk	Moderate risk	Moderate risk	Low risk	Moderate risk
Bian et al., *Medicine* (2021) [5]	Moderate risk	Moderate risk	Low risk	Low risk	Moderate risk	Low risk	Low risk	Moderate risk
Chen et al.,* Biomed Research International* (2020) [6]	Serious risk	Serious risk	Low risk	Low risk	Serious risk	Low risk	Low risk	Serious risk
Suh et al., *Journal of Knee Surgery* (2018) [15]	Moderate risk	Moderate risk	Low risk	Low risk	Moderate risk	Low risk	Low risk	Moderate risk
Palanisamy et al., *Clinical Orthopaedics and Related Research* (2018) [13]	Moderate risk	Moderate risk	Low risk	Low risk	Low risk	Low risk	Low risk	Moderate risk
Kim et al., *Orthopaedics & Traumatology: Surgery & Research* (2018) [7]	Moderate risk	Low risk	Low risk	Low risk	Low risk	Low risk	Low risk	Moderate risk
Li et al., *Official Journal of the Chinese Orthopaedic Association* (2020) [9]	Moderate risk	Moderate risk	Low risk	Low risk	Low risk	Low risk	Low risk	Moderate risk
Wang et al., *BMC Musculoskeletal Disorders* (2021) [11]	Moderate risk	Moderate risk	Low risk	Low risk	Low risk	Low risk	Low risk	Moderate risk

Study characteristics

The 11 studies assessed in this systematic review and pooled analyses included a total of 974 patients [5-15] (Table 2). Two studies were RCTs [9,13], eight were retrospective case-control studies [5-7,9-11,13,15] and one was a prospective, non-randomised study [14]. Nine studies had placebo comparison [5-8,10,12-15], and two used single-dose TXA versus multiple-dose regimes [9,11].

**Table 2:** Study characteristics References: [5-15] TXA: tranexamic acid, RCT: randomised controlled trial

First author, year	Study design	Number of participants	Study period	Age of participants (years)	Male/female	BMI of participants
Luo, 2022 [10]	Retrospective case-control	60	May 2018-May 2019	TXA: 60.3 ± 2.6; control: 60.5 ± 2.7	TXA: 19/11; control: 20/10	TXA: 26.64 ± 2.93; control: 27.61 ± 2.97
Petersen, 2021 [14]	Prospective, non-randomised	52	August 2018-May 2019	TXA group: 51.1 ± 6.9; control: 52.9 ± 7.1	TXA: 16/10; control: 15/11	Not recorded
Bian, 2021 [5]	Retrospective case-control	191	Jan 2016-Aug 2019	Control: 56 ± 6; topical TXA group IV: 54.7 ± 7; TXA group: 56 ± 5	Control: 10/45; topical TXA group IV: 9/55; TXA: 13/59	Control: 24 ± 3; topical TXA group IV: 25 ± 4; TXA: 25 ± 4
Kim, 2021 [8]	Randomised control	73	Oct 2019-Jan 2021	Control: 55.3; TXA: 54.9	Control: 9/27; TXA: 10/27	Control: 26.0; TXA: 26.1
Chen, 2020 [6]	Retrospective cohort	100	August 2018-May 2020	Control: 56.5 ± 10.2; TXA: 58.3 ± 10.4	Control: 22/26; TXA: 20/32	Control: 28.5 ± 4.2; TXA: 27.3 ± 4.0
Ni, 2021 [12]	RCT	100	March 2016-2018	Control: 52.9 ± 1 3.1; TX: 52.5 ± 2.8	TXA group: 10/40; control group: 12/38	TXA: 23.2 ± 1.5; control: 23.5 ± 1.3
Suh, 2018 [15]	Retrospective case-control	30	Control: Oct 2014-Oct 2015; TXA: Nov 2015-Mar 2016	Control: 56 ± 5.7; TXA: 60 ± 5.6	Control: 4/11; TXA: 3/12	Control: 26.1 ± 2.7; TXA: 28.1 ± 3.9
Palanisamy, 2018 [13]	Retrospective case-control	152	Jan 2015-May 2017	Control: 57 ± 6; TXA: 58 ± 5	Control: 9/77; TXA: 7/59	Control: 26 ± 2; TXA: 27 ± 2
Kim, 2018 [7]	Retrospective case-control	150	March 2011-Dec 2016	Control: 55.7 ± 5.5; TXA: 55.0 ± 6.8	Control: 14/61; TXA: 17/58	Control: 26.4 ± 2.6; TXA: 26.3 ± 3.1
Whang, 2021 [11]	Retrospective case-control	54	Jan 2017-Dec 2020	Single dose: 54.63 ± 6.71; two doses: 57.81 ± 5.53; multiple doses: 55.15 ± 6.17	Single dose: 6/12; two doses: 5/13; multiple doses: 8/10	Single dose: 25.73 ± 2.41; two dose: 26.92 ± 2.54; multiple doses: 26.56 ± 1.94
Li, 2020 [9]	Retrospective case-control	56	May 2016-April 2017	Solo group: 58.86 ± 7.696; combined group: 58.63 ± 7.10	Solo group: 10/11; combined group: 10/14	Solo group: 26.59 ± 4.21; combined group: 27.34 ± 5.394

TXA administration

Four of the 11 studies used 1 g TXA IV preoperatively [6,9-11]. Nine of the 11 included studies administered TXA intravenously [5-13] and six involved a multiple-dose regime [6,7,9-12]. Two of the 11 studies did not include a control group but rather compared a single dose to multiple doses [9,11]. This is summarised in Table 3.

**Table 3:** Procedural details of the included studies References: [5-15] TXA: tranexamic acid

First author, year	Study type	Control	Dosing regimen
Luo, 2022 [10]	Retrospective case-control	No TXA	TXA dose 1: 1 g/100 ml, 10 minutes preoperatively; IV TXA dose 2: 3 g/20 ml, on closing; topical TXA dose 3: 1 g/100 ml, 2 hours postoperatively, IV
Petersen, 2021 [14]	Prospective, non-randomised	No TXA	TXA dose 1: 1 g, 5 minutes preoperatively, route not stated
Bian, 2021 [5]	Retrospective case-control	No TXA	IV group: 10 mg/kg in 100 ml saline, 10 minutes preoperatively; topical group: 10 mg/kg; timing: intraoperatively; route: topical and subcutaneously to the osteotomy site
Kim, 2021 [8]	Randomised control	No TXA	TXA dose 1: 2 g (500 mg/5 ml), 10 minutes preoperatively, IV
Chen, 2020 [6]	Retrospective cohort	No TXA	TXA dose 1: 1 g, preoperatively; IV TXA dose 2: 1 g intraoperatively, topical to the osteotomy site
Ni, 2021 [12]	Randomised control	No TXA	TXA dose 1: 50 mg/kg, 10 minutes before tourniquet deflation, IV
Suh, 2018 [15]	Retrospective case-control	No TXA	TXA dose 1: 2 g/20 ml, postoperatively, haemovac line
Palanisamy, 2018 [13]	Retrospective case-control	No TXA	TXA dose 1: 2 g, 10 minutes preoperatively; IV TXA dose 2: 2 g, 3 hours after the first dose, IV
Kim, 2018 [7]	Retrospective case-control	No TXA	TXA dose 1: 10 mg/kg, preoperatively; IV TXA dose 2: 10 mg/kg, 6 hours postoperatively; IV TXA dose 3: 10 mg/kg, 24 hours postoperatively, IV
Wang, 2021 [11]	Retrospective case-control	No control	All groups: 1 g, 15-30 minutes preoperatively, IV. Two- and three-dose only: 1 g, 6 hours postoperatively, IV. Three-dose only: 1 g, per day until postoperative day (POD) 3, IV
Li, 2020 [9]	Retrospective case-control	No control	All groups: 1 g/100 ml, preoperatively, IV. Combined group only: 2 g/20 ml, on closure, drainage tube

Total blood loss

**Figure 2:**
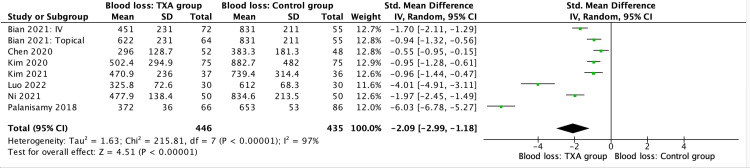
Total blood loss results, table and forest plot of the pooled estimated standardised mean differences (SMDs) References: [5-8,10,12,13] TXA: tranexamic acid

In the six TXA versus placebo studies reporting on the total blood loss, the TXA group had a pooled, estimated SMD in blood loss of -2.37 (95% CI -3.67, -1.07; P = 0.0004) (Figure 2).

Hb decrease

**Figure 3:**
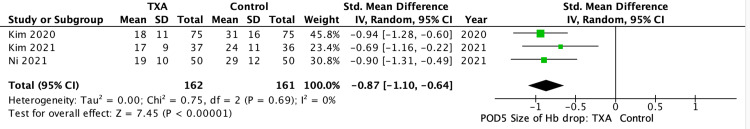
Haemoglobin decrease results 1, table and forest plot of pooled estimated standardised mean differences (SMDs). Results grouped by data availability of postoperative days [7,8,12]. TXA: tranexamic acid

**Figure 4:**
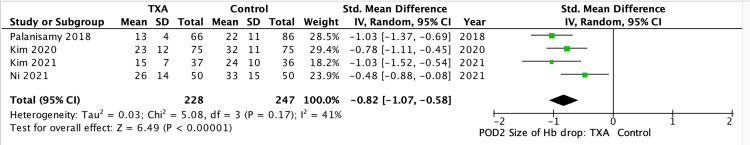
Haemoglobin decrease results 2, table and forest plot of pooled estimated standardised mean differences (SMDs). Results grouped by the data availability of postoperative days [7,8,12,13]. TXA: tranexamic acid

**Figure 5:**
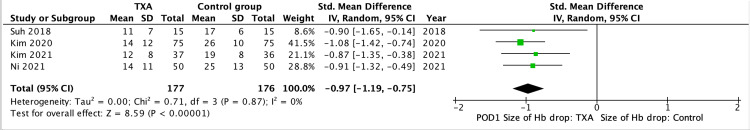
Haemoglobin decrease results 3, table and forest plot of pooled estimated standardised mean differences (SMDs). Results grouped by data availability of postoperative days [7,8,12,15]. TXA: tranexamic acid

Hb decrease was measured on different postoperative days. Four studies reported on POD 1, with a pooled, estimated SMD of -0.97 (95% CI -1.19, -0.75; P < 0.00001). POD 2 saw a pooled, estimated SMD of -0.82 (95% CI -1.07, -0.58; P < 0.00001) of the same four studies. Three studies reported on POD 5 Hb decrease with a pooled estimated SMD of -0.87 (95% CI -1.10, -0.64; P < 0.00001) (Figures 3-5).

Drain output

**Figure 6:**
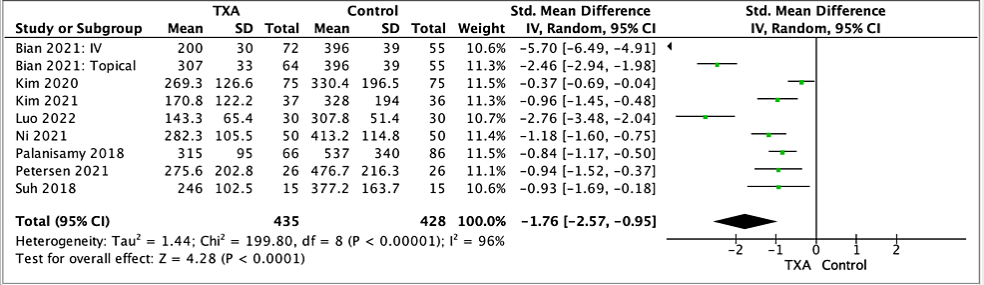
Drain output results, table and forest plot of pooled estimated standardised mean differences (SMDs) References: [5,7,8,10,12-15] TXA: tranexamic acid

Eight studies reported on the drain output with a pooled, estimated SMD of 1.76 (95% CI -2.57, -0.95; P < 0.0001) (Figure 6).

Wound complications

**Figure 7:**
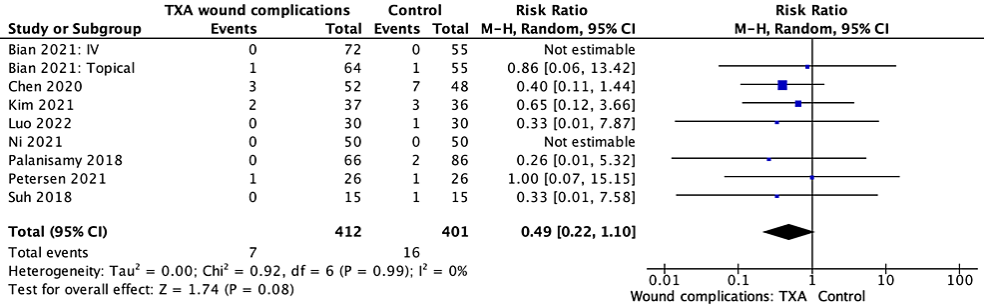
Wound complications results, table and forest plot of pooled estimated standardised mean differences (SMDs) References: [5,6,8,10,12-15] TXA: tranexamic acid

The prevalence of wound complications was not significantly significant; nine studies saw a pooled estimated SMD of 0.49 (95% CI 0.22, 1.10; P = 0.08) (Figure 7).

Need for transfusion

**Figure 8:**
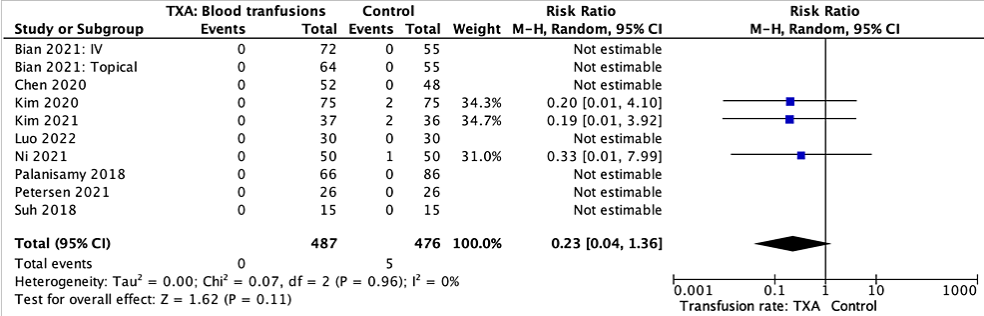
Need for transfusion results, table and forest plot of pooled estimated standardised mean differences (SMDs) References: [5-8,10,12-15] TXA: tranexamic acid

Similarly, there was no statistical significance between groups when regarding the rate of blood transfusion. Ten studies reported on the requirement for transfusion, with a pooled estimated SMD of 0.23 (Figure 8). 

MOWHTO is known to be a procedure that risks high blood loss. This is due to a variety of reasons, including intraoperative exposure of highly vascularised cancellous bone [11]. In addition, MOWHTO typically involves torniquet use to optimise surgical view of the operative field [15]. Studies have demonstrated an abnormal increase in fibrinolytic activity in patients undergoing torniquet procedures, which has the potential to perpetuate bleeding for at least 15 minutes [16]. Perioperative blood loss increases the risk of haematoma formation around the incision site, anaemia and blood transfusion rates [11,17]. Orthopaedic literature has reported that blood transfusion is an independent predictor for increased length of inpatient stay [18]. In addition, transfusion requirement is of particular significance in light of the recent reduction in the availability of blood due to limited stock resulting in the necessity to use alternative means when appropriate, including TXA [19]. A previous systematic review has confirmed that TXA can reduce the relative risk of requiring a blood transfusion by 45% in MOWHTO [20].

In our study, a decrease in total blood loss was seen in all 11 studies. This result can be explained by the mechanism of TXA; acting as a competitive inhibitor, TXA binds lysine sites on plasminogen, preventing the enzymatic breakdown of fibrin meshwork [21]. Previous literature corroborated this effect; Ma et al. (2021) found that TXA reduced blood loss, decreased Hb and reduced wound complications [22]. Similarly, TXA has successfully reduced blood loss in an RCT focusing on total knee replacements [23].

Due to its antifibrinolytic effect, there remains understandable concern that TXA has the potential to increase rates of deep vein thrombosis (DVT). However, it has been demonstrated that preoperative TXA does not increase the rates of either DVT or pulmonary embolism (PE) in total knee replacements [24]. The study mentioned was prospective and single-blinded with TXA infusion post-total knee replacement. Follow-up occurred at three months postoperatively, and no thromboembolic events were observed in the TXA group. Although an interesting result, further investigation is recommended, largely due to both a small sample size and absence of control group. A further trial has demonstrated that the rate of DVT is not increased by TXA whether used IV or s/c for TKA [25]. A 2021 meta-analysis of studies between 1976 and 2020 also disproved the theory, demonstrating that TXA did not increase thromboembolic events and did reduce bleeding-associated mortality [26]. However, it did mention a high degree of variation between both follow-up and postoperative care between included studies. There remain patient groups where TXA is advised to be used with caution, including renal impairment due to excretion by glomerular filtration [27]. Cases of ophthalmological disturbance following TXA usage have been reported in the literature, including altered colour perception and retinal artery occlusion [28]. However, it has been emphasised that further literature is required in this area and the relationship between the two is not considered directly causal; a future systematic review of this effect would likely be of value. Currently, TXA is approved and considered safe for clinical use in post-partum haemorrhage, acute traumatic blood loss, cardiothoracic surgery and bleeding disorders [21].

While none of the studies included in this review reported the effect of TXA use on the length of stay, the senior authors in their high-volume osteotomy practice have found that the length of stay in patients undergoing MOWHTO has significantly decreased with the use of TXA. This is likely due to reduced rate of transfusions. While the literature surrounding MOWHTO on TXA effect on the length of stay is lacking, there is strong evidence from TKA studies that we can extrapolate from [29].

## Conclusions

MOWHTO is a popular early surgical intervention for OA, but it risks blood loss. This systematic review and meta-analysis has determined that TXA is a safe and effective therapy to reduce blood loss in MOWHTO surgery. This result could demonstrate a substantial improvement in postoperative outcomes, which consequently impact patient the length of stay and thus overall NHS expenditure. However, to establish this effect, we believe that larger, randomised trials would be of value to the literature. In addition, there remains opportunity for review articles determining both the impact of intraoperative TXA use upon overall inpatient length of stay and lesser reported side effects of TXA, including ophthalmological disturbances.

## Appendices

Annex A 

**Table 4:** Search strategies by database NCBI: ational Center for Biotechnology Information, CINAHL: Cumulative Index of Nursing and Allied Health Literature

Database	Search strategy
NCBI	((osteotomy) OR (high tibial osteotomy) OR (hto) OR (knee) OR (proximal tibia)) AND ((tranexamic acid) OR (txa))
Embase	((osteotomy or high tibial osteotomy or hto or proximal tibia) and (tranexamic acid or txa))
CINAHL	((osteotomy) OR (high tibial osteotomy) OR (hto) OR (proximal tibia) OR (knee)) AND ((tranexamic acid) OR (txa))

## References

[1] Wallace IJ, Worthington S, Felson DT (2017). Knee osteoarthritis has doubled in prevalence since the mid-20th century. Proc Natl Acad Sci U S A.

[2] Zhang H, Fan Y, Wang R (2020). Research trends and hotspots of high tibial osteotomy in two decades (from 2001 to 2020): a bibliometric analysis. J Orthop Surg Res.

[3] Capella M, Gennari E, Dolfin M, Saccia F (2017). Indications and results of high tibial osteotomy. Ann Jt.

[4] (2022). NHS England » Tackling the elephant in the room. https://www.england.nhs.uk/blog/tackling-the-elephant-in-the-room/.

[5] Bian J, Deng B, Wang Z (2021). Comparison of topical and intravenous tranexamic acid for high tibial osteotomy: a retrospective study. Medicine (Baltimore).

[6] Chen DS, Zhu JW, Wang TF, Zhu B, Feng CH (2020). Tranexamic acid is beneficial to patients undergoing open-wedge high tibial osteotomy. Biomed Res Int.

[7] Kim KI, Kim HJ, Kim GB, Bae SH (2018). Tranexamic acid is effective for blood management in open-wedge high tibial osteotomy. Orthop Traumatol Surg Res.

[8] Kim MS, Koh IJ, Sung YG, Park DC, Ha WJ, In Y (2021). Intravenous tranexamic acid has benefit for reducing blood loss after open-wedge high tibial osteotomy: a randomized controlled trial. J Clin Med.

[9] Li S, Lu Q, Guo X, Zhang M, Miao Z, Luo D, Liu P (2020). Intravenous combined with topical tranexamic acid administration has no additional benefits compared with intravenous administration alone in high tibial osteotomy: a retrospective case-control study. Orthop Surg.

[10] Luo W, Fu X, Huang JM, Wu J, Ma XL (2022). Efficacy and safety of intravenous combined with topical administration of tranexamic acid in reducing blood loss in opening wedge high tibial osteotomy procedure: a retrospective case-control study. Acta Orthop Traumatol Turc.

[11] Wang Z, Huang Q, Liu L (2021). Dose tranexamic acid reduce blood loss associated with simultaneous bilateral distal tibial tubercle-high tibial osteotomy?. BMC Musculoskelet Disord.

[12] Ni J, Liu J, Zhang J, Jiang J, Dang X, Shi Z (2021). Tranexamic acid is beneficial for blood management of high tibial osteotomy: a randomized controlled study. Arch Orthop Trauma Surg.

[13] Palanisamy JV, Das S, Moon KH, Kim DH, Kim TK (2018). Intravenous tranexamic acid reduces postoperative blood loss after high tibial osteotomy. Clin Orthop Relat Res.

[14] Petersen W, Bentzin M, Bierke S, Park HU, Häner M (2022). Use of tranexamic acid in medial open wedge high tibial osteotomy. Arch Orthop Trauma Surg.

[15] Suh DW, Kyung BS, Han SB, Cheong K, Lee WH (2018). Efficacy of tranexamic acid for hemostasis in patients undergoing high tibial osteotomy. J Knee Surg.

[16] Klenerman L, Chakrabarti R, Mackie I, Brozovic M, Stirling Y (1977). Changes in haemostatic system after application of a tourniquet. Lancet.

[17] Kalra S, Thilagar B, Khambaty M, Manjarrez E (2021). Post-operative anemia after major surgery: a brief review. Curr Emerg Hosp Med Rep.

[18] Bou Monsef J, Boettner F (2014). Blood management may have an impact on length of stay after total hip arthroplasty. HSS J.

[19] (2023). NHSBT amber alert and the use of tranexamic acid in surgery — Royal College of Surgeons. https://www.rcseng.ac.uk/news-and-events/news/archive/nhsbt-amber-alert-and-the-use-of-tranexamic-acid-in-surgery/#:~:text=In%20line%20with%20these%20measures,perioperative%20care%20theatre%20checklist%20process.

[20] Ker K, Beecher D, Roberts I (2013). Topical application of tranexamic acid for the reduction of bleeding. Cochrane Database Syst Rev.

[21] Cai J, Ribkoff J, Olson S, Raghunathan V, Al-Samkari H, DeLoughery TG, Shatzel JJ (2020). The many roles of tranexamic acid: an overview of the clinical indications for TXA in medical and surgical patients. Eur J Haematol.

[22] Ma J, Lu H, Chen X, Wang D, Wang Q (2021). The efficacy and safety of tranexamic acid in high tibial osteotomy: a systematic review and meta-analysis. J Orthop Surg Res.

[23] Veien M, Sørensen JV, Madsen F, Juelsgaard P (2002). Tranexamic acid given intraoperatively reduces blood loss after total knee replacement: a randomized, controlled study. Acta Anaesthesiol Scand.

[24] Zohar E, Fredman B, Ellis M, Luban I, Stern A, Jedeikin R (1999). A comparative study of the postoperative allogeneic blood-sparing effect of tranexamic acid versus acute normovolemic hemodilution after total knee replacement. Anesth Analg.

[25] Zekcer A, Del Priori R, Tieppo C, da Silva RS, Severino NR (2016). Topical vs. intravenous administration of tranexamic acid in knee arthroplasty and prevalence of deep venous thrombosis: a randomized clinical trial. J Vasc Bras.

[26] Taeuber I, Weibel S, Herrmann E (2021). Association of intravenous tranexamic acid with thromboembolic events and mortality: a systematic review, meta-analysis, and meta-regression. JAMA Surg.

[27] Yang QJ, Jerath A, Bies RR, Wąsowicz M, Pang KS (2015). Pharmacokinetic modeling of tranexamic acid for patients undergoing cardiac surgery with normal renal function and model simulations for patients with renal impairment. Biopharm Drug Dispos.

[28] Al Shaharani AM, Al Ghamdi TA (2020). Central retinal vein occlusion associated with the use of tranexamic acid. Saudi J Ophthalmol.

[29] D'Souza R, Duncan C, Whiting D (2021). Tranexamic acid is associated with decreased transfusion, hospital length of stay, and hospital cost in simultaneous bilateral total knee arthroplasty. Bosn J Basic Med Sci.

